# Automated multiparametric localization of prostate cancer based on B-mode, shear-wave elastography, and contrast-enhanced ultrasound radiomics

**DOI:** 10.1007/s00330-019-06436-w

**Published:** 2019-10-10

**Authors:** Rogier R. Wildeboer, Christophe K. Mannaerts, Ruud J. G. van Sloun, Lars Budäus, Derya Tilki, Hessel Wijkstra, Georg Salomon, Massimo Mischi

**Affiliations:** 1 Lab of Biomedical Diagnostics, Department of Electrical Engineering, Eindhoven University of Technology, De Rondom 70, 5612 AP Eindhoven, The Netherlands; 2 Department of Urology, Amsterdam University Medical Centers, University of Amsterdam, Meibergdreef 9, 1105 AZ Amsterdam, The Netherlands; 3 Martini-Clinic – Prostate Cancer Center, University Hospital Hamburg Eppendorf, Martinistraße 52, 20246 Hamburg, Germany; 4 Department of Urology, University Hospital Hamburg-Eppendorf, Martinistraße 52, 20251 Hamburg, Germany

**Keywords:** Prostate cancer, Machine learning, Ultrasonography, Elasticity imaging techniques, Contrast media

## Abstract

**Objectives:**

The aim of this study was to assess the potential of machine learning based on B-mode, shear-wave elastography (SWE), and dynamic contrast-enhanced ultrasound (DCE-US) radiomics for the localization of prostate cancer (PCa) lesions using transrectal ultrasound.

**Methods:**

This study was approved by the institutional review board and comprised 50 men with biopsy-confirmed PCa that were referred for radical prostatectomy. Prior to surgery, patients received transrectal ultrasound (TRUS), SWE, and DCE-US for three imaging planes. The images were automatically segmented and registered. First, model-based features related to contrast perfusion and dispersion were extracted from the DCE-US videos. Subsequently, radiomics were retrieved from all modalities. Machine learning was applied through a random forest classification algorithm, using the co-registered histopathology from the radical prostatectomy specimens as a reference to draw benign and malignant regions of interest. To avoid overfitting, the performance of the multiparametric classifier was assessed through leave-one-patient-out cross-validation.

**Results:**

The multiparametric classifier reached a region-wise area under the receiver operating characteristics curve (ROC-AUC) of 0.75 and 0.90 for PCa and Gleason > 3 + 4 significant PCa, respectively, thereby outperforming the best-performing single parameter (i.e., contrast velocity) yielding ROC-AUCs of 0.69 and 0.76, respectively. Machine learning revealed that combinations between perfusion-, dispersion-, and elasticity-related features were favored.

**Conclusions:**

In this paper, technical feasibility of multiparametric machine learning to improve upon single US modalities for the localization of PCa has been demonstrated. Extended datasets for training and testing may establish the clinical value of automatic multiparametric US classification in the early diagnosis of PCa.

**Key Points:**

*• Combination of B-mode ultrasound, shear-wave elastography, and contrast ultrasound radiomics through machine learning is technically feasible.*

*• Multiparametric ultrasound demonstrated a higher prostate cancer localization ability than single ultrasound modalities.*

*• Computer-aided multiparametric ultrasound could help clinicians in biopsy targeting.*

## Introduction

With more than an estimated 164,000 new diagnoses in the USA [1] and almost 450,000 in Europe [2], prostate cancer (PCa) remains the most frequently occurring non-skin malignancy in Western men in 2018. Unfortunately, after prostate-specific antigen (PSA) serum level testing and/or digital rectal examination, the standard diagnostic approach strongly relies on a 10- to 12-core systematic biopsy [3]. Aside from complications associated with this procedure [4], high levels of underdiagnoses and overtreatment have been reported [5]. Given the strong clinical demand for reliable imaging that enables targeted biopsy, recent years have shown promising advances in multiparametric magnetic resonance imaging (mpMRI). Whereas individual modalities of MRI are not considered sufficiently accurate in PCa diagnosis, mpMRI leverages the combination of these modalities through scoring according to the Prostate Imaging Reporting and Data System (PI-RADS) [6]. The 2019 guidelines of the European Association of Urology recommend the use of a pre-biopsy mpMRI in the diagnostic pathway. However, aside from some inherent limitations of MRI (e.g., its high cost, limited availability, and impracticality for bedside use), such scoring systems are known to exhibit a slow learning curve and are at risk of high operator disagreement [7].

Another potential candidate for PCa imaging is ultrasound (US), which is cost-effective, widely available, and practical. Even though US modalities such as shear-wave elastography (SWE) and dynamic contrast-enhanced ultrasound (DCE-US) have shown promising results, targeted biopsy with US techniques still is not superior over systematic biopsy [8]. However, to date, a multiparametric US approach has been scarcely investigated [9]. The rationale for a multiparametric approach (i.e., combining information from complementary biomarkers such as tissue texture, elasticity or perfusion to image a notoriously multifocal and heterogeneous disease like PCa [10]) applies to both MRI and US. On top of that, the use of quantitative features known as radiomics is gaining attention [11]. Radiomics quantifies the spatial representation of tissue in an image such as heterogeneity or asymmetric enhancement by locally extracting textural and statistical features from the (parametric) images. In this work, we strived to combine the information from different modalities as well as their radiomics for image-based diagnosis of PCa. To examine the potential of such an approach, we employed machine learning technology by means of a random forest to optimally combine the underlying parameters. A random forest forms the core of a computer-aided diagnosis algorithm that combines all information into a single multiparametric image for the clinician to review [12].

In the classifier, inputs from B-mode US, SWE, and DCE-US are considered [13]. Although B-mode US by itself is not a suitable option for PCa imaging, biopsy guidelines highly recommend targeting of suspicious hypoechoic lesions [5]. As for SWE, tissue stiffness is regarded as a strong indicator of malignancy [14]. Recent studies have demonstrated its usefulness for the detection of PCa [15–17]. DCE-US, in which contrast agents are employed to visualize the vascularity, allows the assessment of tissue perfusion and contrast dispersion [18]. In fact, it was shown that quantification of the contrast agent kinetics by contrast ultrasound dispersion imaging (CUDI) allows the estimation of parameters reflecting the characteristics of angiogenic (micro)vasculature [19–21]. Whereas DCE-US images primarily represent vascular tissue characteristics, SWE images are related to the cell density and collagen deposition in the tissue [22, 23]. Therefore, being complementary in nature, it can be hypothesized that their combination leads to an increased diagnostic potential. In a recently published study, perfusion- and dispersion-related DCE-US parameters were already successfully combined in a machine learning approach [24].

This work validates a proposed random forest–based classifier in a leave-one-patient-out fashion, both pixel-wise and region-wise. Furthermore, the correlations among different features were investigated and their individual and combined importance for the localization of (clinically significant) PCa was evaluated.

## Materials and methods

### Data acquisition

At the Martini Clinic Prostate Cancer Centre (University Hospital Hamburg Eppendorf, Germany), 50 men with biopsy-confirmed PCa referred for radical prostatectomy (RP) underwent a multiparametric US procedure. Only patients with a PSA level below 20 ng/mL, a prostate volume of < 80 mL, and no indication of extracapsular invasion were included in the study; patients with contra-indication for DCE-US or previous PCa therapy were excluded. Institutional review board approval was acquired and all participants signed an informed consent. Of them, 48 men underwent RP and were included in the study. The patient characteristics are listed in Table 1. Each patient received a B-mode, SWE, and 2-min DCE-US recording of the apex, mid, and base section of the gland. The examinations were performed manually, with an Aixplorer® ultrasound scanner (SuperSonic Imagine) equipped with a SE12-3 endocavity probe. For the DCE-US recordings, a 2.4-mL bolus of SonoVue® (Bracco) was intravenously administered. DCE-US was performed in “Gen” contrast-specific, low-mechanical-index mode; SWE images were obtained with minimal pre-compression and after a few-second stabilization period. This work is related to a clinical trial on multiparametric ultrasound (i.e., under registration number NCT03091231) and more information on the clinical workflow can be found in a previously published protocol paper [25].

**Table 1:** Characteristics of the patient group

Parameter	Value
Number of patients, *n*	48
Age, median (IQR)	65 (58–70) years
TRUS volume, median (IQR)	40 (34–49) mL
PSA, median (IQR)	7.7 (5.3–10.4) ng/mL
Radical prostatectomy index Lesion Gleason score, *n*	
3 + 3 = 6	1 (2.1%)
3 + 4 = 7	30 (62.5%)
4 + 3 = 7	6 (12.5%)
> 4 + 3 = 7	11 (22.9%)

*IQR*, interquartile range

### Histopathological examination

After resection, the RP specimens were histopathologically examined. The annotated PCa regions were used to reconstruct a 3D model of the prostate and its lesions [26]. This model was subsequently digitally cross-sectioned at the apex, mid gland, and base to be matched to the imaging planes, allowing for direct US histopathology comparison [27]. Taking into account registration inaccuracy, a maximum of one unambiguously malignant and one unambiguously benign region of interest (ROI) were delineated in the B-mode image to serve as labeled ground truth for training and validation. The ROIs were drawn such that the number of malignant and the number of benign pixels, as well as those originating in the peripheral zone (PZ) and transition zone (TZ), were in balance.

### Algorithm structure

**Fig. 1:**
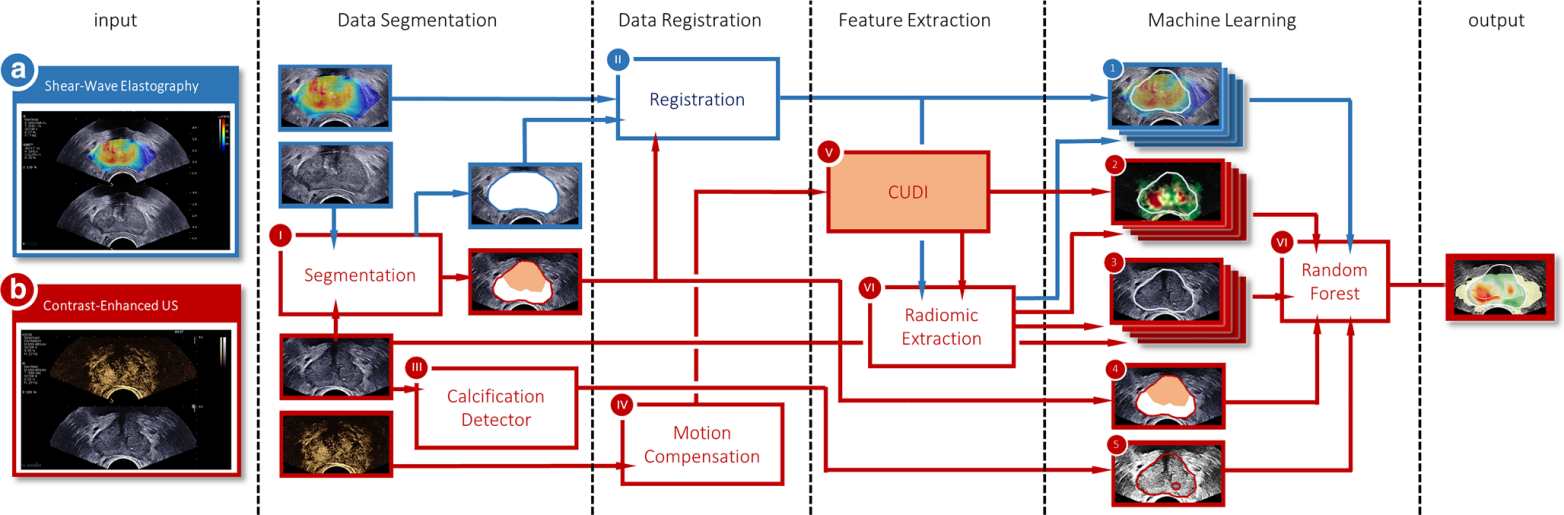
Schematic overview of the proposed classification framework, with information from shear-wave elastography and contrast-enhanced ultrasound recording shown in blue and red, respectively

An overview of the proposed method is shown in Fig. 1 and comprises (A) prostate segmentation, (B) data registration, (C) feature extraction, and (D) multiparametric classification. Testing and validation of the model is discussed in the last section of this “Materials and methods” section.

#### Prostate segmentation

Firstly, the prostate is located and delineated in each modality. To this end, we employed an automated deep learning–based TRUS segmentation algorithm on the side-view fundamental B-mode images of both the SWE and DCE-US acquisition [28, 29]. For DCE-US, the prostate position during wash-in (i.e., at 30 s) was used as a reference. Automatically, the prostate images were also zonally segmented, labeling pixels belonging to either the PZ or TZ for further use in the classification algorithm. The deep learning–based segmentations were checked by both an engineer and a urologist with 4 years of experience in TRUS imaging. Aside from prostate segmentation, a detection algorithm was designed to outline calcifications in the B-mode images. Calcifications were identified by high-valued regions in the fundamental-mode image after convolution with 2D Gaussian kernels having an empirically chosen standard deviation of ~ 0.6 mm and ~ 1.8 mm, thus detecting hyperechoic spots with diameters of approximately 1.2 and 3.6 mm. The purpose of calcification detection was to prevent false-positive readings due to elevated stiffness of calcified regions.

#### Data registration

As the proposed method aims at pixel-specific classification, a pixel-to-pixel match between the different US modalities is required. Again, the 30-s fundamental view image of the DCE-US recording was chosen as a reference. The SWE data were elastically registered to this image based on the segmented contours. Moreover, motion compensation was applied to the DCE-US video by rigid registration of every 5th frame to the reference position; the registration of the intermediate frames was performed by interpolating the translation-rotation matrix.

#### Imaging feature extraction

The proposed classifier includes a two-step feature extraction. First, model-based blood flow features are retrieved from the DCE-US imaging. The model-based feature extraction serves two purposes: on the one hand, physically meaningful parameters with known correlation to PCa are estimated and, on the other hand, the dimensionality of the DCE-US is reduced to 2D, matching the SWE and grayscale image prior to texture analysis. Secondly, radiomic features are extracted from the resulting model-based feature maps as well as the SWE and grayscale image.

The model-based feature extraction was based on CUDI, a family of quantification methods that estimate underlying physical quantities of a DCE-US recording related to perfusion and dispersion [19–21, 30, 31]. A total of 12 DCE-US features were extracted for every pixel, which are listed in Table 2. In CUDI, the spreading of contrast through the prostate is regarded as a convective-dispersive process, which can be quantified by assessing the evolution of contrast over time. The contrast velocity (*v*), dispersion (*D*), and Péclet (Pe) number were estimated through local system identification [21]. Alternatively, the local degree of dispersion can also be quantified by the similarity in contrast behavior among pixels. This was quantified either by spatiotemporal correlation (*r*) [30, 31] or spectral coherence (*ρ*) [20]. In addition, we fitted the contrast curves in a single pixel by a modified local density random walk model, enabling us to estimate the mean transit time (*μ*), the dispersion-related parameter (*κ*), and the area under the contrast curve (*α*) [19]. Finally, also heuristic parameters such as the wash-in time (WIT), appearance time (AT), peak intensity (PI), and peak time (PT) were extracted.

**Table 2:** Diagnostic performance of parameters

Modality	Parameter	Pixel-wise		Region-wise	
		≥ Gleason 3 + 3 = 6	> Gleason 3 + 4 = 7	≥ Gleason 3 + 3 = 6	> Gleason 3 + 4 = 7
DCE-US	Pe, Péclet number (−)	0.63	0.63	0.67	0.69
	*v*, velocity (mm/s)	0.66	0.70	0.69	0.76
	*D*, dispersion (mm^2^/s)	0.52	0.52	0.56	0.57
	*r*, spatiotemporal correlation (−)	0.66	0.70	0.69	0.76
	*ρ*, spectral coherence (−)	0.64	0.65	0.66	0.68
	*κ*, dispersion parameter (s^−1^)	0.59	0.62	0.62	0.67
	*μ*, mean transit time (s)	0.61	0.69	0.64	0.71
	*α*, area under TIC (a.u.)	0.56	0.58	0.50	0.53
	WIT, wash-in time (s)	0.61	0.69	0.64	0.72
	PT, peak time (s)	0.64	0.71	0.63	0.68
	AT, arrival time (s)	0.57	0.60	0.57	0.56
	PI, peak intensity (a.u.)	0.61	0.65	0.57	0.65
SWE	*E*, Young’s modulus	0.62	0.67	0.62	0.73
B-mode	G, gray level	0.54	0.58	0.53	0.58
Classifier	Multiparametric score	0.70	0.78	0.75	0.90

The rationale for the use of radiomic features is that not only pixel values but also local spiculation, heterogeneity, and granularity are widely considered as important biomarkers of cancer. Moreover, asymmetric patterns in perfusion or elasticity regardless of the pixel values are also seen as indicative of malignancy. To take into account intra-prostate asymmetry, as well as relatively high parameter values, we introduced the parameter value relative to the median parameter value per image as a feature. Likewise, to quantify parameter heterogeneity, we extracted the entropy of the parameter distribution in a circular kernel around the pixel of interest. A multiscale approach was adopted, using heuristic kernel radii of ~ 1 mm, ~ 2 mm, and ~ 3 mm. In addition, the parameter variance was calculated in a ~ 2-mm kernel.

#### Automated multiparametric combination

Multiparametric combination of the features was achieved through machine learning based on a random forest algorithm. A random forest is an ensemble of independently trained decision trees, which vote together on the final classification score [32]. Having a branch-like structure of decision nodes, single-classification trees classify a sample by a series of decisions based on the input variables. Node by node, the tree structure is grown by evaluating for which feature (a subset of) the labeled training instances can be most effectively separated in terms of their class. Subsequently, the robustness of a random forest is established by growing each tree using another random subset of the training samples [33].

In this work, we enforced the first split to be based on the zonal location (either PZ or TZ), as it is established that tissue stiffness [34] and the influx of contrast agents [35] differ substantially between zones. Then, a random forest was grown consisting of 1000 trees using 1/1000th of the training set with replacement. To promote generalizability, six random training patients were completely discarded prior to growing each tree. The cross-entropy of labels within the nodes was adopted as the splitting criterion and the tree depth was at most 50 nodes. Pixels containing calcifications were omitted in the training phase as they might obscure the underlying tissue type. The final multiparametric score, ranging from − 1 to 1, was defined by the ratio between the number of malignant and benign classifications among the trees in the random forest. After classification, outliers were removed from the multiparametric images by assigning the median multiparametric score in a 15-pixel region (~ 2.5 mm), corresponding to approximately half the radius of clinically significant PCa [36].

### Validation and statistical analysis

The classifier was validated in a leave-one-patient-out cross-validation procedure, in which each patient is tested using 1 classifier that is trained on the data of the remaining patients. The performance was assessed by computing the area under the receiver operating characteristics curve (ROC-AUC) of the parameter values or the multiparametric score, both in a pixel-wise and a region-wise fashion. In the latter approach, a ROI was characterized by its mean parameter value or multiparametric score. Differences between distributions were statistically assessed with a Wilcoxon rank sum test [37]. Throughout this work, *p* values of < 0.05 and < 0.005 are defined to describe significant and highly significant differences among groups, respectively.

## Results

### Correlation among radiomics

**Fig. 2:**
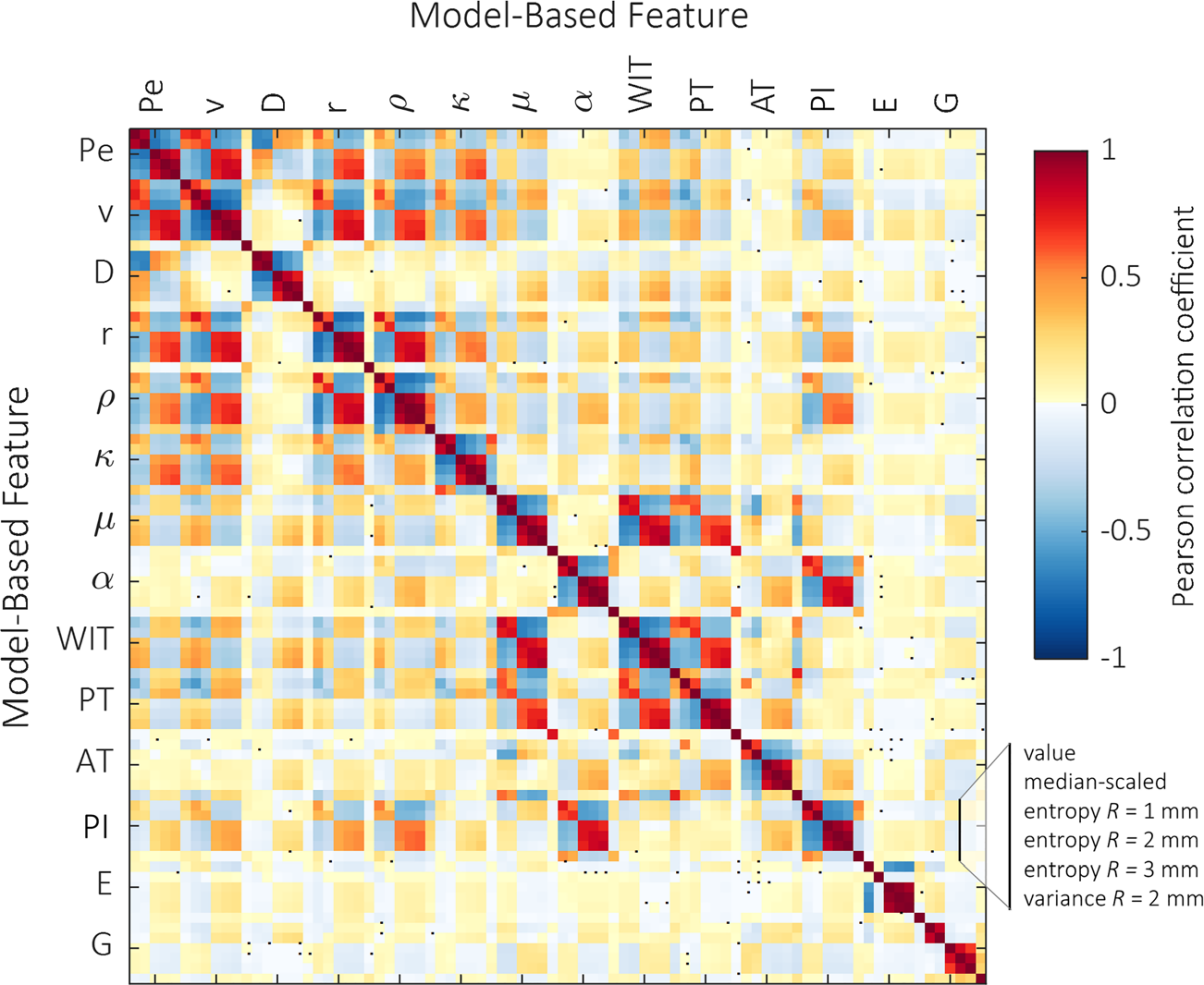
Correlation matrix of the derived radiomics in terms of the linear Pearson correlation coefficient; correlations that are not significantly (*p* > 0.05) reflected by a linear correlation are indicated by a black square

Figure 2 depicts the correlations between radiomics in a correlation matrix. Strong positive and negative correlations are color-coded in red and blue, respectively. Features from the same analysis typically exhibit high correlation. In addition, especially *μ* and WIT as well as *α* and PI seem related. The low correlation between Young’s modulus (*E*), gray levels (*G*), and DCE-US features is an indication that B-mode, SWE, and DCE-US are indeed complementary.

### Classification performance

**Fig. 3:**
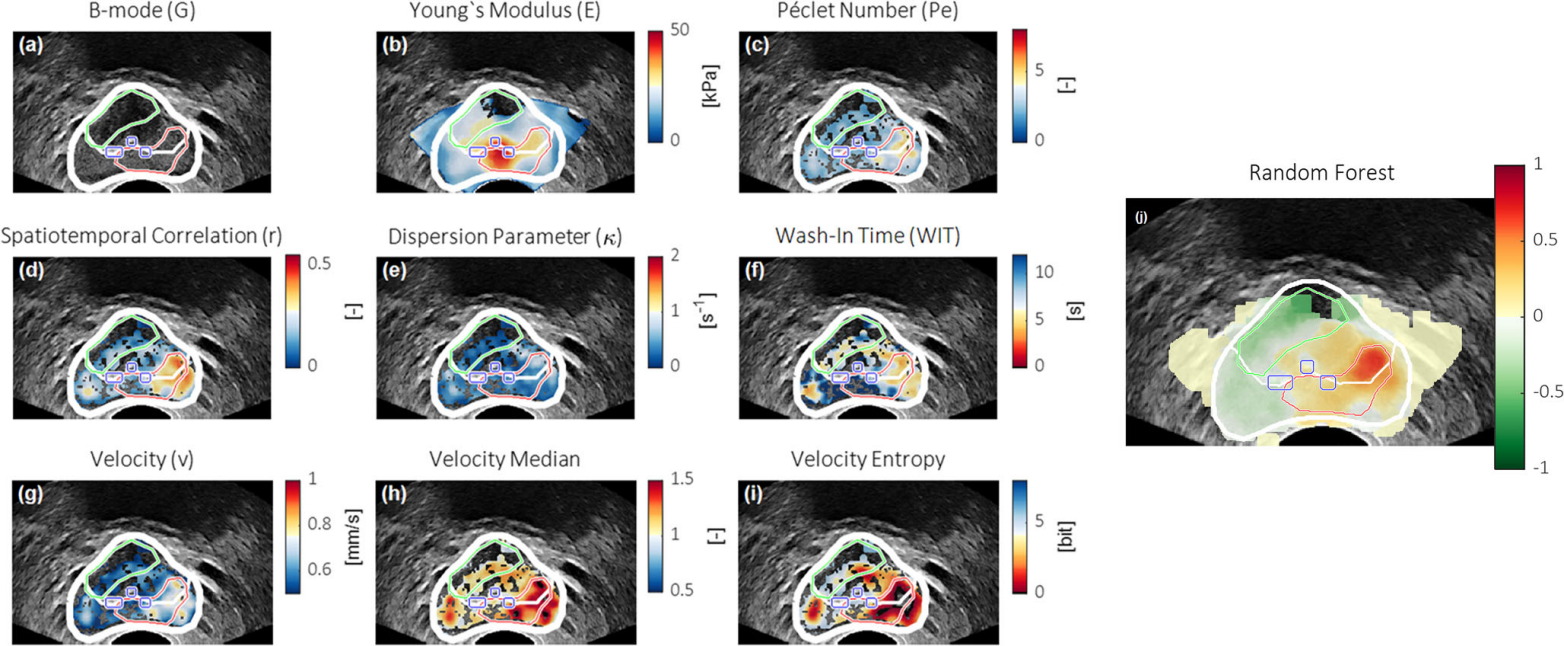
Image plane example, showing the B-mode (**a**), Young’s modulus (SWE) (**b**), Péclet number (**c**), spatiotemporal correlation (**d**), dispersion-related parameter (**e**), wash-in time (**f**), velocity (**g**), velocity relative to image median (**h**), 2-mm entropy of velocity (**i**), and resulting multiparametric map (**j**). In each map, the prostate and zonal segmentations are depicted in white, the calcifications are encircled in blue, and histopathologically confirmed malignant and benign ROIs are indicated in red and green, respectively

Figure 3 illustrates the power of the proposed multiparametric analysis, showing a number of single-parametric maps alongside the multiparametric image obtained in a patient with a left-apical 4 + 5 = 9 tumor. The segmentation, zonal boundary, detected calcifications, and annotated ROI locations are indicated as well. The single- and multiparametric results across the entire dataset are presented in Table 2. Multiparametric radiomic-based classification yields a region-wise ROC-AUC of 0.75 and 0.90 for PCa and significant PCa versus benign regions, respectively. In our dataset, binary ROI classification (i.e., with a positive multiparametric score referring to malignancy and a negative to benign tissue) would lead to 32 (27%) benign regions erroneously classified as sPCa and 1 (3.3%) sPCa lesion as benign.

In comparison, applying the classifier on only the radiomic features for the best-performing DCE-US parameter (i.e., contrast velocity, *v*) resulted in a region-wise ROC-AUC of 0.71 and 0.84 for PCa and sPCa, respectively. The classification performance can thus be partly attributed to the use of radiomics and partly to multiparametric combination. With the use of non-contrast features, only region-wise ROC-AUCs of 0.58 for PCa and 0.65 for sPCa were achieved.

### Feature relevance

**Fig. 4:**
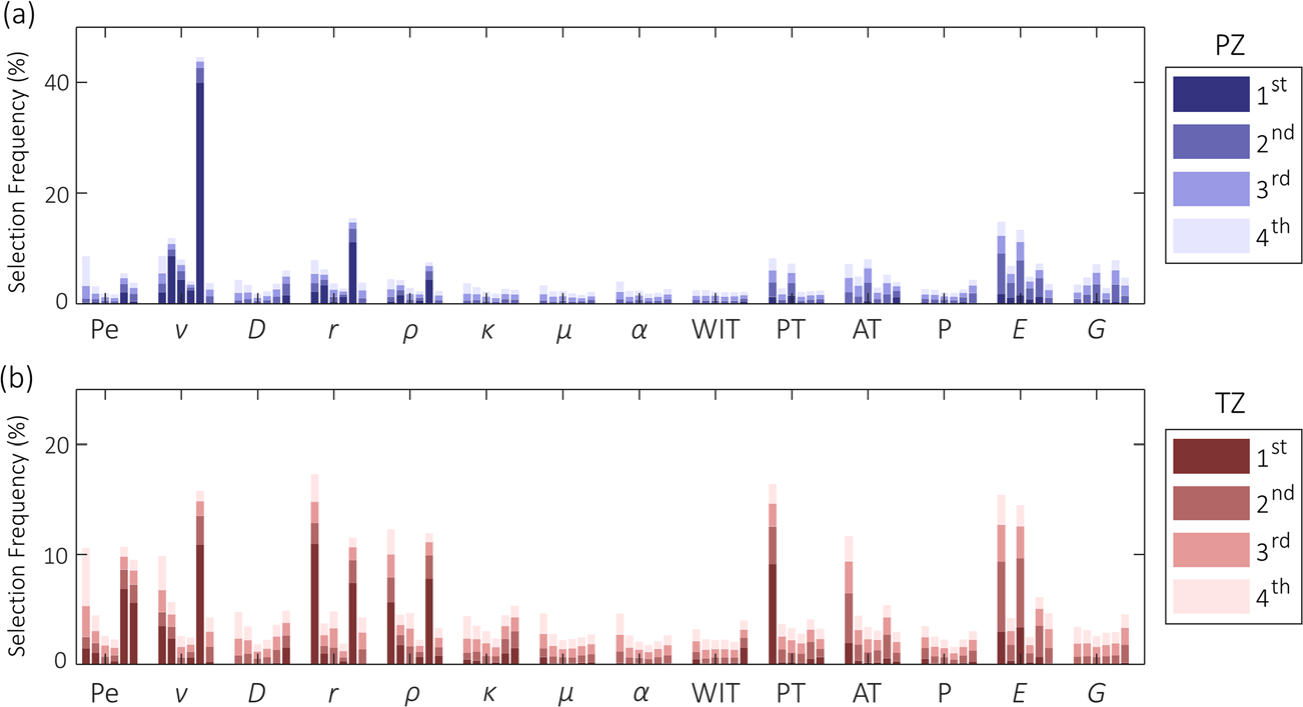
Overview of the frequency at which radiomics are selected for the highest-order branches among all trees in the forest. Radiomics are grouped according to the model-based parameters

The relative relevance of different parameters is assessed by examining which parameters are selected for the first, second, etc. decision nodes in the trees of the random forest. Figure 4 presents the most prominent parameters. Based on this data, it seems that in particular the combination between *v*, *r*, and *E* is favored. In the TZ, also *ρ* and PT are relevant parameters. In terms of radiomics, mostly the parametric value itself and the large-kernel entropy are selected.

### Relation to cancer grade

**Fig. 5:**
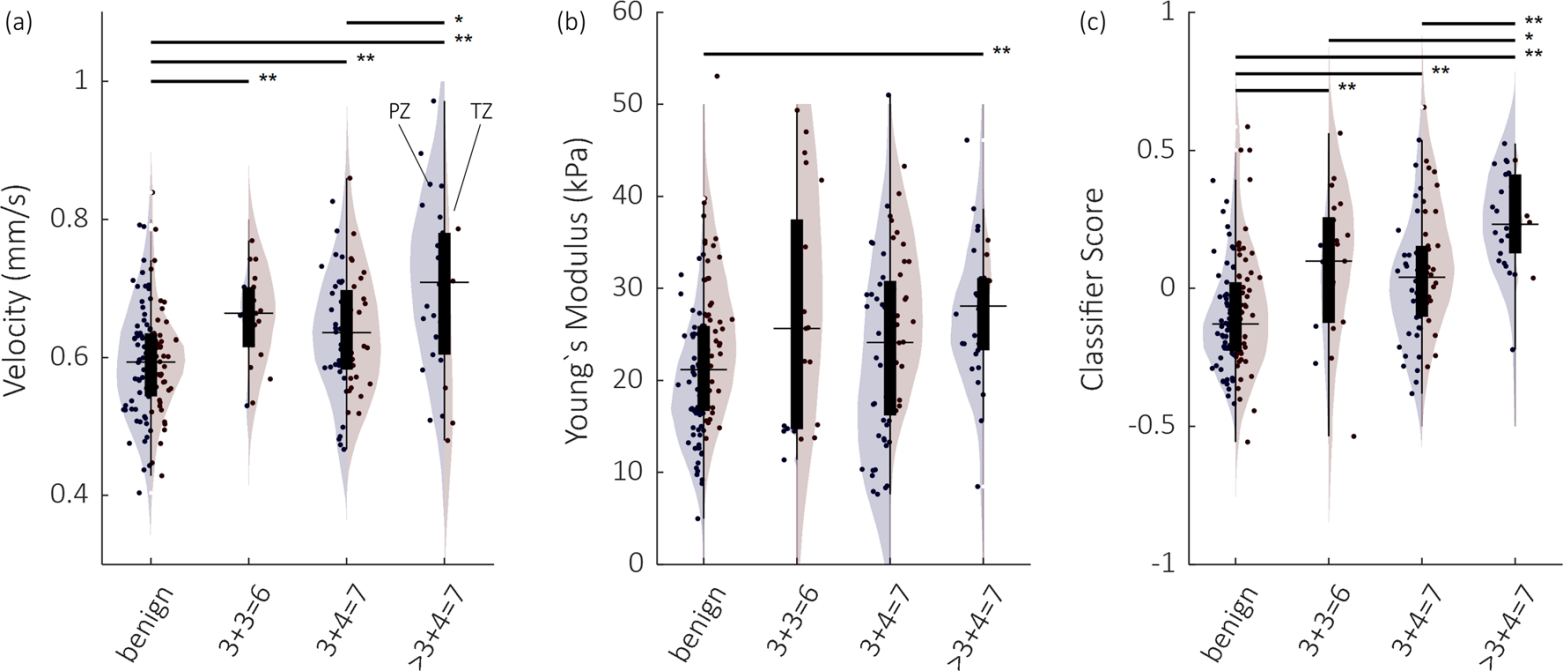
Overview of the parameter values and classifier score for the velocity (**a**), Young’s modulus (*E*) (**b**), and the multiparametric classifier score (**c**). Individual regions are represented by a bullet. The violin plots represent the group distribution in the PZ (left, blue) and TZ (right, red). Significant and highly significant differences according to a Wilcoxon rank sum test are indicated with a single asterisk and double asterisks

To assess the degree to which parameters and the multiparametric score correspond to cancer aggressiveness, Fig. 5 shows how the mean values per ROI are distributed for different Gleason groups and prostate zones (i.e., PZ and TZ). Both SWE-derived Young’s modulus and the best-performing DCE-US parameter (i.e., *v*) are depicted alongside the final multiparametric score. Significant and highly significant differences are indicated with a single asterisk and double asterisks, respectively. It should be emphasized that healthy TZ tissue is generally stiffer than PZ tissue [34], as evidenced in Fig. 5, hampering the analysis of TZ and PZ regions as a single group.

## Discussion

In this work, we report on the development of a random forest–based classifier for multiparametric classification of PCa based on co-registered B-mode, shear-wave elastography, and contrast-enhanced ultrasound. Aside from model-based parameters, radiomics are introduced in the classifier framework to extract additional information from the parametric maps. The ROC-AUC, validated in a leave-one-patient-out cross-validation fashion, shows a region-wise improvement from 0.76 of the best-performing individual parameter, *v*, to a multiparametric 0.90 for significant Gleason > 3 + 4 PCa. A similar improvement is achieved using a pixel-wise approach. The improvement is partly the result of the radiomic extraction and partly of the multiparametric combination.

The random forest classifier is a powerful tool for classification that allows for the integration of a large range of (radiomic) features and generates an intuitive multiparametric score. The frequency at which parameters are being selected for classification (see Fig. 4) substantiates the multiparametric hypothesis, favoring a combination of a perfusion-related (i.e., *v*, PT), dispersion-related (i.e., *r*, *ρ*), and elastographic (i.e., *E*) parameters. This is in line with earlier work that only included DCE-US parameters, reporting that model-based parameters that are related to different underlying biomarkers combine most effectively [24]. In addition, the selected parameters differ substantially between the PZ and TZ. This might be due to the anatomic or physiological differences between zones; however, it could also be a result of less robust parameter estimation farther away from the probe (i.e., in the TZ), due to the increasing impact of attenuation and shadowing. This stresses the need for adequate zonal segmentation in the proposed framework, here obtained through deep learning [28, 29].

The multiparametric score is shown to scale with tumor Gleason grade, with significant differences between benign, insignificant, and significant disease. Several definitions of clinically significant prostate cancer are used in the literature; due to the limited amount of 3 + 3 disease in this RP-validated study and the distinction between Gleason 3 + 4 = 7 and 4 + 3 = 7 being strongly associated with PCa prognosis [38], we report on both the identification of ≥ 3 + 3 and > 3 + 4 PCa. Our results (Fig. 5) show no significant difference between 3 + 4 PCa and the small group of 3 + 3 PCa. This might be partly explained due to a bias in the 3 + 3 group, with tumors being disproportionately large for clinicians to decide upon RP as a treatment instead of active surveillance and, thus, for inclusion in the presented study.

Furthermore, only a few radiomic features were introduced in this research. Many more have been proposed in the literature, including morphological, intensity-based, texture-based, and statistics-based features [39–43]. Alternatively, novel model-based features could be considered. For example, velocity vector field entropy [44] and viscoelasticity [23, 45] have shown promise as markers for PCa in DCE-US and SWE. Even though the quality of the input images remains dependent on the operator recording the SWE and CEUS acquisitions [46, 47], the use of automatically generated single multiparametric images might reduce interobserver variability compared with cognitive reading of a large ensemble of parametric maps.

Compared with other research, a meta-analysis of SWE and DCE-US has reported ROC-AUCs of 0.90 [15] to 0.91 [16] and 0.83 [48], respectively. It should be emphasized that these results are based on systematic biopsy, known for its systematic and random errors [49], as a reference standard. Furthermore, these scores were obtained by cognitive reading of images and videos rather than an automatic pixel value–based approach like in this study, which might hamper comparison. The value of multiparametric images for cognitive reading, either as stand-alone tool or combined with the source images, remains to be investigated.

Despite the performance gains obtained using the proposed method, some malignant ROIs were still missed and some benign ROIs were wrongly classified as malignant by the algorithm. In the future, immunohistochemical techniques [50, 51] might elucidate more on the nature of the false readings. There are indications that (co-occurring) prostatitis or BPH might be responsible for false positives, as these diseases are known to also promote angiogenesis [52, 53]. Qualitative inspection of the false negatives revealed that these were indeed invisible to the naked eye on all US modalities. Future analysis of tumors that are missed on all US imaging modalities might potentially direct us towards new parameters or radiomic features contributing to the multiparametric classification.

Furthermore, this study was conducted in a single center, where a dataset of 50 patients presenting biopsy-proven PCa was collected in order to have RP specimens as histopathological ground truth for PCa localization. As a result, ROC-AUCs were calculated for the separation of benign and malignant ROIs. Prospective, multicenter, targeted biopsy-based studies might eventually confirm the diagnostic value of the machine learning classification presented in this work in a more varied patient group [54]. Another limitation is the 2D nature of this approach, requiring the acquisition of three planes per patient for every modality. In a clinical setting, the use of more planes per patient would reduce the risk of missing out-of-plane tumors at the cost of an increased procedure time. However, as 3D SWE and DCE-US have recently been introduced [55, 56], expansion to three dimensions can be envisaged.

In conclusion, we demonstrated the feasibility of a multiparametric classifier to improve upon single US modalities for the localization of PCa. This is in line with recently published work on multiparametric US for the identification of malignant and benign breast lesions [57]. We aim to further extend the dataset, so that the classification approach can be expanded to more radiomics and features. Once the performance is consolidated, we believe that a three-dimensional approach might bring clinical adoption closer within reach.

## Abbreviations

CUDI: Contrast ultrasound dispersion imaging
DCE-US: Dynamic contrast-enhanced ultrasound
MRI: Magnetic resonance imaging
PCa: Prostate cancer
PI-RADS: Prostate imaging reporting and data system
PSA: Prostate-specific antigen
PZ: Peripheral zone
ROC-AUC: Area under the receiver operating characteristics curve
ROI: Region of interest
RP: Radical prostatectomy
SWE: Shear-wave elastography
TZ: Transition zone
US: Ultrasound
